# Assessing the Opportunity for an Accelerated Access Pathway for Health Canada Priority Review Drugs: A Comparative Analysis with Ontario’s FAST Pilot Program

**DOI:** 10.3390/curroncol33090517

**Published:** 2026-08-29

**Authors:** Catherine Y. Lau, Arif Mitha, Allison Wills

**Affiliations:** 1 Independent Researcher, 158 Front Street E, Toronto, ON M5A 0K9, Canada; cathy.lau456@gmail.com; 2 20Sense Corp., Toronto, ON M4J 1G2, Canada; amitha@20sense.ca

**Keywords:** drugs, cancers, patients, access, Health Canada, Project Orbis, Funding Accelerated for Specific Treatments (FAST), Priority Review (PR), Notice of Compliance with conditions (NOC/c), Canada’s Drug Agency (CDA-AMC), pan-Canadian Pharmaceutical Alliance (pCPA), accelerated access

## Abstract

Background: Timely public reimbursement of innovative medicines remains a challenge in Canada despite expedited regulatory review pathways. This study evaluated whether an accelerated reimbursement pathway, similar to Ontario’s Funding Accelerated for Specific Treatments (FAST) for oncology drugs approved through Project Orbis could improve access for therapies approved through Health Canada’s Priority Review (PR) pathway, extending to indications beyond oncology. Methods: Health Canada drug submissions completed between 2021 and 2025 were reviewed to characterize PR, Notice of Compliance with conditions (NOC/c), and Project Orbis. Drug submissions completed in 2022 were selected for detailed analysis. Drug review and approval process data were compiled from Health Canada (HC), Canada’s Drug Agency (CDA-AMC), the pan-Canadian Pharmaceutical Alliance (pCPA), Ontario government, and manufacturer sources. Time from Health Canada Notice of Compliance (NOC) to Ontario public listing was compared for Orbis, PR non-Orbis, and FAST therapies. Results: Among drugs approved in 2022, mean time from NOC to Ontario listing was 625.29 ± 365.80 days for PR non-Orbis and 604.18 ± 283.92 days for Orbis. FAST therapies were listed in 229.22 ± 140.50 days, approximately 60% faster than Orbis and PR non-Orbis. Half of PR approvals in 2022 were not associated with Project Orbis and were therefore ineligible for existing accelerated reimbursement pathways. Conclusions: The Ontario FAST program is associated with substantially shorter times to public listing for novel oncology medicines. Extending a similar accelerated access pathway to therapies approved through PR could improve timely and equitable patient access in Canada.

## Simple Summary

Canadians often wait years to receive public funding for new medicines, even after approval by Health Canada. Ontario recently introduced a pilot program called FAST to accelerate public funding for certain new cancer medicines, but this approach is limited to a small group of drugs reviewed under a program called Project Orbis. This study examined whether a similar expedited approach could be expanded to include medicines that receive Health Canada’s Priority Review designation, which is granted to therapies that address serious diseases and unmet medical needs, in oncology and other indications. We found that medicines funded through Ontario’s pilot program reached public reimbursement about 60% faster than comparable Priority Review medicines that were not eligible for accelerated access programs. Expanding accelerated reimbursement to include these medicines could improve timely and equitable access to innovative treatments for Canadian patients.

## 1. Introduction

### 1.1. Canada’s Time to Drug Access

Timely and equitable access to innovative medicines that demonstrate superior efficacy and safety is essential for improving patient outcomes and healthcare quality. However, Canada’s public reimbursement system for novel therapies is widely regarded as complex and slow. Compared with other Organisation for Economic Co-operation and Development (OECD) countries, Canada ranks among the slowest for patient access to new therapies, with public reimbursement typically occurring more than 1.5 years after regulatory approval [1]. This slow pace reflects Canada’s multilayered drug reimbursement process, which involves federal, pan-Canadian, and provincial reviews. These processes often lack transparency and are difficult for manufacturers, clinicians, patients, and other stakeholders to navigate [2,3].

### 1.2. Health Canada’s Priority Review Process

In response to the urgent need for effective treatments for patients facing critical illnesses, regulatory authorities around the world introduced Priority Review processes during the 1990s [4,5,6,7]. On 13 December 1996, Health Canada (HC) implemented its own policy for the ‘Priority Review of Drug Submissions’ [6]. This initiative significantly shortened the review timeline for certain drug applications, reducing it from the standard 300 days to 180 days.

The Priority Review (PR) process in Canada is specifically intended for drugs that aim to treat, prevent, or diagnose serious, life-threatening, or severely debilitating medical conditions [6]. To qualify, the medication must provide a substantial benefit or introduce a major therapeutic advancement over currently available treatments. This pathway is open to both new drug submissions (NDS) and applications for new indications of existing drugs, called supplemental new drug submissions (SNDS).

From 2021 to 2025, the number of PRs granted for NDS and SNDS in Canada as a total of all approvals ranged from 11% to 22% per year [8]. These approvals span a variety of conditions including cancers, rare diseases, cardiovascular conditions, immune-related conditions, and others [8].

Regarding the effectiveness of PR, a recent analysis suggests that oncology therapies reviewed under PR up to the end of 2024 have higher therapeutic values than those approved through the standard or Notice of Compliance with conditions (NOC/c) review pathways [9]. Other evaluations of the PR pathway found a lack of consistent therapeutic value for drugs approved in 2016 and earlier [10,11]; however, these findings may not apply to more recently approved therapies as therapeutic composition of new drugs approved under PR has shifted since the end of 2020 [8]. It has also been highlighted that the overall time required for patients to access new treatments may be similar for both priority and standard reviews. An analysis comparing the time from regulatory approval to pan-Canadian listing found no significant difference between drugs reviewed under priority versus standard processes [12].

Following the introduction of the PR process, Health Canada established another accelerated pathway known as the NOC/c [13]. This process allows for market approval based on promising preliminary evidence in situations where there is a serious condition with unmet medical needs. It requires sponsors to undertake confirmatory clinical trials after initial approval, and until the outcomes of the studies are available, clinicians are using the medications without confirmed efficacy and safety. The review time for NOC/c submissions is set at 200 days. Canada’s Drug Agency (CDA-AMC) allows manufacturers to file HTA submissions as early as 180 days before the anticipated date of decision by Health Canada, with data from 2025 showing that 75% of submissions elected to use this process (although the length of time of these submissions prior to NOC is not reported), while 25% did not [14]. In 2023, CDA-AMC introduced the Time-Limited Recommendation (TLR) category [15], and the pan-Canadian Pharmaceutical Alliance (pCPA) established a temporary access process (pTAP) for NOC/c products [16]. These processes cover primarily oncology drugs [17], and uptake has been minimal with only three products leveraging the TLR/pTAP processes in the almost 3 years since launch, largely due to the narrow eligibility criteria for entry into the programs [18,19].

### 1.3. The FDA’s Project Orbis Initiative and Its Impact on Access to Oncology Drugs in Canada

#### 1.3.1. Project Orbis

In May 2019, the U.S. Food and Drug Administration (FDA) Oncology Center of Excellence launched Project Orbis [20], an initiative designed to allow multiple countries to review promising new oncology drugs or indications simultaneously. The objective is to accelerate global review timelines for therapies targeting life-threatening diseases, and files can be selected for Project Orbis based on a combination of a breakthrough designation, impressive results, and a high unmet need. Health Canada and the Australian Therapeutic Goods Administration were co-founding partners of Project Orbis, together with the FDA [21,22], and were later joined by several other international regulatory agencies. Health Canada has been a highly active participant, reviewing nearly 80 new drugs or indications through this pathway by the end of 2025 [8,21]. In Canada, Project Orbis submissions are expected to meet the criteria of one of Health Canada’s expedited review pathways, either PR or NOC/c [21]. A recent study found that drugs approved via the Orbis pathway, especially those also receiving Priority Reviews, demonstrate greater clinical benefit compared to non-Orbis drugs [9]. Similar comparisons have not been undertaken for non-Orbis products. Project Orbis has notably expedited regulatory review times and narrowed the gap between U.S. and Canadian submission timelines. However, a study evaluating drug access in England, Scotland, and Canada from 1 May 2019, to 1 November 2023, found that the overall impact on reducing time to patient access remains unchanged [23].

#### 1.3.2. Canadian Accelerated Access Pathways for Oncology Drugs Approved via Project Orbis

Recognizing the significant clinical impact of new oncology drugs and indications approved through the Project Orbis initiative, in October 2025 both the Government of Ontario (Ontario) and the pCPA initiated targeted accelerated access pilot programs. Ontario launched the Funding Accelerated for Specific Treatments (FAST) program, a three-year pilot for select Project Orbis oncology drugs which provides early public funding as soon as a positive final health technology assessment (HTA) recommendation is granted by CDA-AMC [24]. Participation in the FAST pilot is not automatic for all eligible therapies [24]. The Ontario Ministry of Health assesses each file individually and may choose not to select a drug for FAST for reasons including complex negotiations, eligibility for another accelerated funding pathway, or where funding through FAST is not considered to be in the public interest [24]. Funding is available while the pCPA and drug manufacturer negotiate the drug price [24]. The payment mechanism for the drugs patients take in this pilot project has not been publicly disclosed [25]. As of 15 June 2026, nine therapies had been listed via the FAST program. Early reports suggest that for these first nine therapies, time to listing was achieved 60% faster than what is typical for oncology drugs [26]. In parallel, the pCPA introduced the Early Negotiation Process (ENP) pathway, which offers expedited negotiations for oncology drugs approved through Project Orbis [27]. The negotiation process begins when an HTA body, either CDA-AMC or Quebec’s Institut national d’excellence en santé et en services sociaux (INESSS), accepts a submission, which is earlier than the process for a standard negotiation [27]. The number of files that have gone through this pathway is not publicly available. Both programs have been strongly praised by patient advocacy groups and industry [28,29]. It is, however, important to note that these programs are currently limited to oncology drugs.

#### 1.3.3. Gaps for Other Drugs Approved Under Health Canada’s Priority Review Pathway

While Project Orbis offers one category of drugs for accelerated access, this eligibility criterion is limited in scope to a subset of oncology therapies. Comparable reimbursement pathways are notably absent for Health Canada’s existing expedited PR pathway, which is a longstanding mechanism to accelerate approvals for drugs with a high unmet need, in both oncology and non-oncology. The threshold for clinical efficacy and safety is high for drugs that qualify for PR, and these drugs often treat serious diseases where no alternatives exist or where no significant clinical improvement over current therapies has been demonstrated. Without an accelerated access process, these drugs often face delays of two to three years before reaching patients through Canada’s public drug plans [1], which contributes to inequities in drug access for patients across the country [28].

This article evaluates opportunities to establish a more timely and equitable drug access framework for Canadian patients who are candidates for therapies approved through the Health Canada PR pathway. It assesses whether an accelerated access pathway, modelled on the Ontario FAST pilot program, could improve access for PR therapies by comparing the access timelines of therapies listed under FAST against those approved via PR, which are currently not eligible for existing accelerated access pathways.

## 2. Methods

The data search was conducted between 1 April 2026, and 16 June 2026.

Health Canada data for completed drug submissions from 2021 to 2025 were reviewed [8]. Approved files for new active substances or new indications for previously approved drugs were included. Biosimilars were removed. PR was defined as any file that was reviewed by Health Canada as PR and resulted in a Notice of Compliance (NOC). Any files that were reviewed by HC as PR and resulted in an NOC/c were considered NOC/c.

Drug submissions completed by Health Canada in 2022 were examined further. The year 2022 was selected so that a full view of drug access timelines would be available in the analysis, given the average 2-year timeline from NOC to first public listing in Canada. Using Submission Under Review data from the Health Canada website (Excel SUR list as of 30 April 2026) [8], each file was examined to be identified as follows: the date of approval, the disease category, the HC review pathway at approval, Project Orbis or not. A descriptive analysis was performed to determine file volumes by review pathway and disease category, and evaluate the overlap between the three HC review pathways (standard, NOC/c and PR) and Project Orbis. Additional descriptive and timeline information was obtained for each drug from pCPA and CDA-AMC websites, with an analysis cutoff date of 15 June 2026.

Ontario listing information for the selected drugs was obtained from multiple sources, starting with the Exceptional Access Program (EAP) [30] and Ontario Government press releases [31,32], followed by patient group websites [33], the drug manufacturer, and the drug’s Cancer Care Ontario Eligibility forms using the patient support program eligibility end date [34,35,36], with an analysis cutoff date of 15 June 2026. If no listing date was found from these sources, the drug was deemed not to be listed. Vaccines were excluded from the Ontario listing exercise. An additional data validation for the Ontario listing dates was carried out by an independent consultant, using the same methodology. Data were then grouped for analysis, Orbis and PR non-Orbis. (Table S1). A time-to-listing analysis was conducted from the Health Canada NOC date to the Ontario listing date for all drugs within each data group.

Therapies listed under Ontario’s FAST pilot program were identified from the Government of Ontario’s website and drug manufacturer press releases [37,38,39,40], with an analysis cutoff date of 15 June 2026. Additional descriptive and timeline information was obtained for each FAST drug from Health Canada, CDA-AMC, and pCPA websites. Ontario listing dates were obtained from the Ontario Ministry of Health’s Drug Programs Delivery (HPDD) Notice emails [41] (Table S2). A time-to-listing analysis was then conducted from Health Canada NOC to Ontario listing date for each file.

Time-to-listing comparisons were then analyzed for each group (Orbis; PR non-Orbis) from the 2022 approval data, and the FAST drugs, using Mean ± standard deviation (SD) (95% confidence interval [CI]) and Median (interquartile range [IQR]) in Excel statistical programs. Parametric Paired T Test was used to compare the differences in time from NOC to Ontario listing and to CDA-AMC final recommendation. Whisker plots from Excel were used to compare listing times. Data extraction was led by one author and validated by two others. Data analysis was led by one author and verified by two others. Discrepancies were resolved by discussion amongst the authors until consensus was reached.

## 3. Results

**Figure A1:**
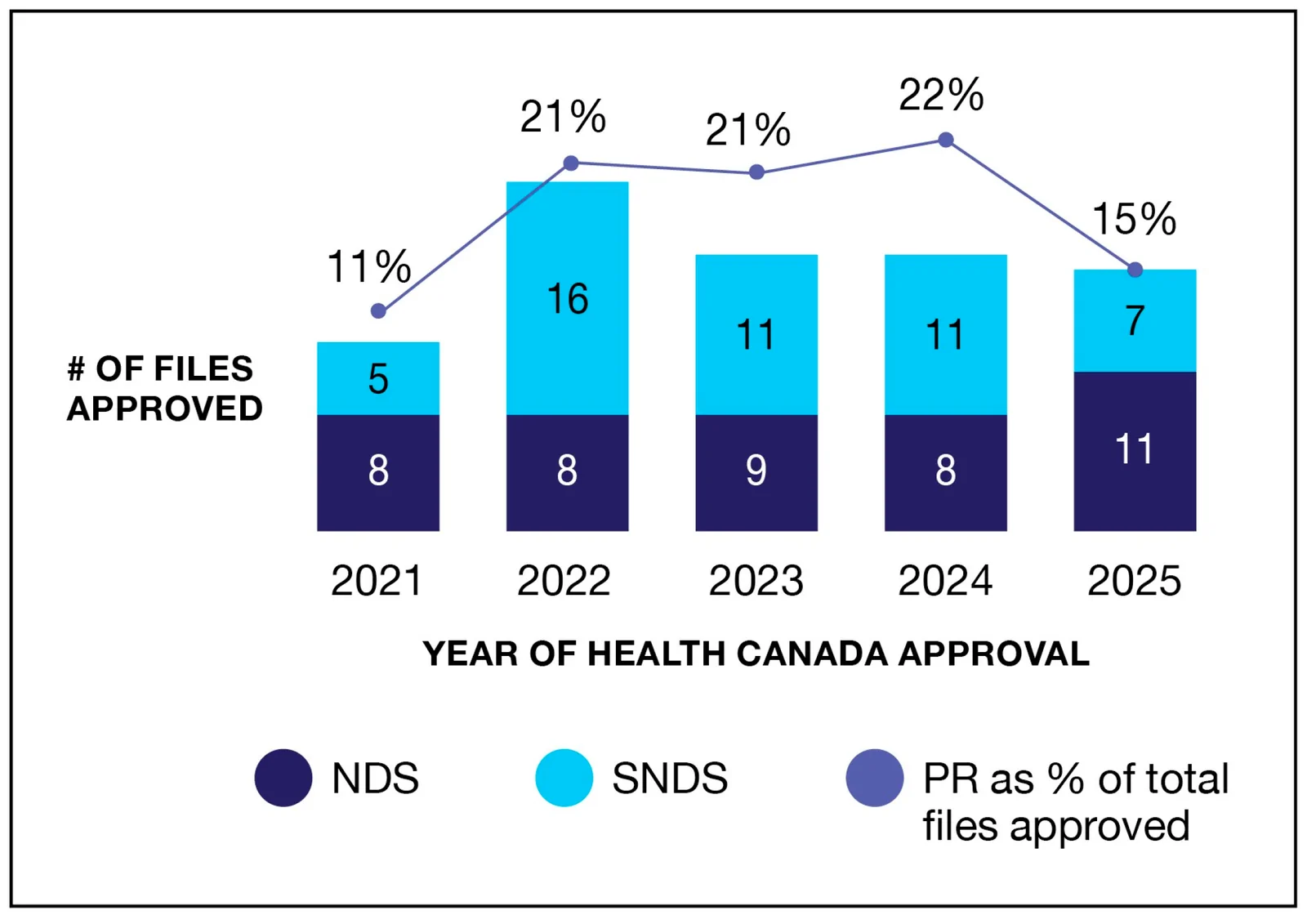
Health Canada Priority Review files approved by year. NDS = New Drug Submission; PR = Priority Review; SNDS = Supplemental New Drug Submission.

Over the 5-year period from 2021 to 2025, the proportion of PR approvals granted for new drugs and new indications ranged from 11% to 22% annually with an average of 18% of PR files per year. Among these PR approvals, 47% of files were NDS; 53% were SNDS (Figure A1).

**Figure 1:**
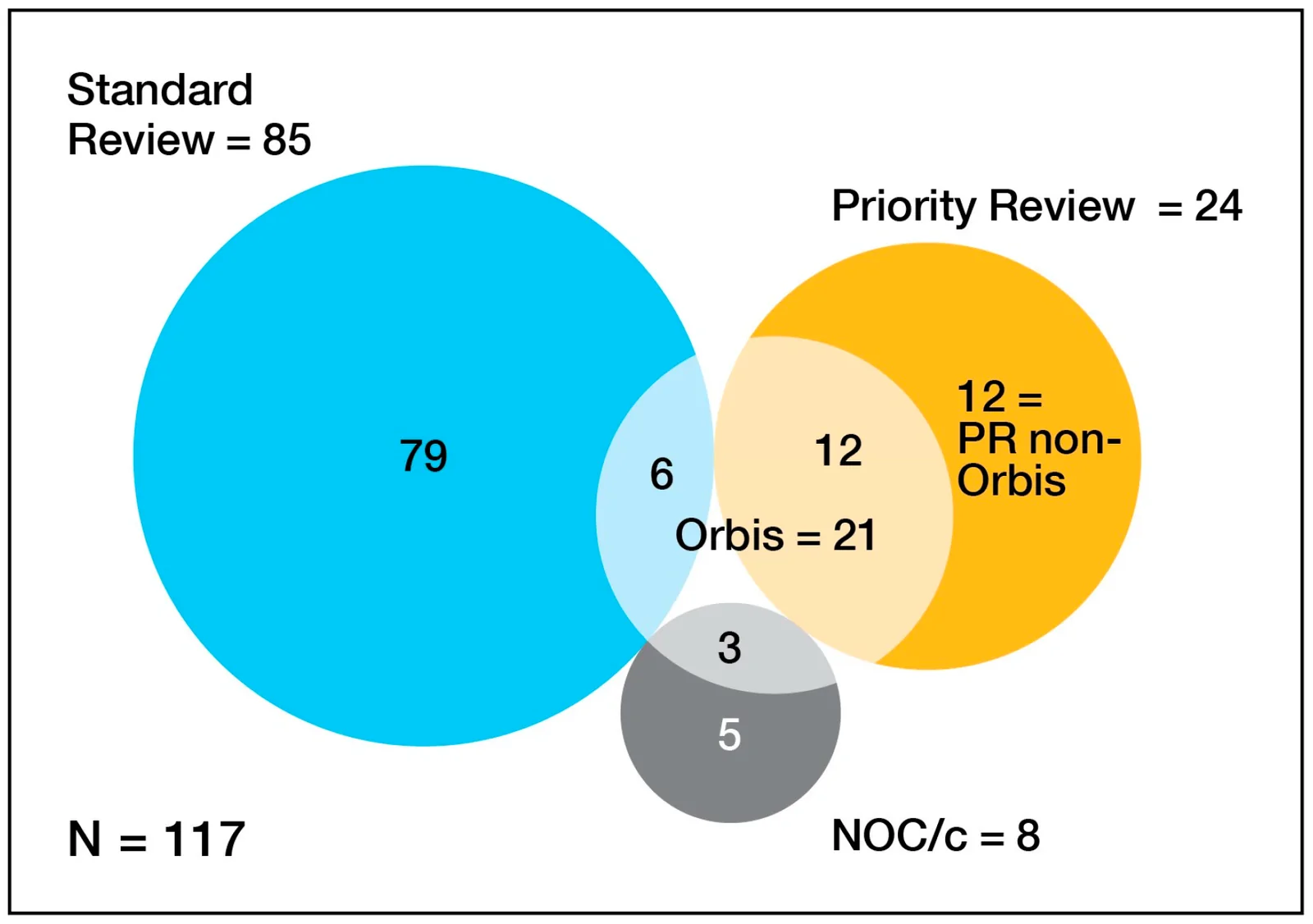
Overlap between Health Canada files approved in 2022 by review pathway & Project Orbis. NOC/c = Notice of Compliance with conditions; PR = Priority Review.

**Figure A2:**
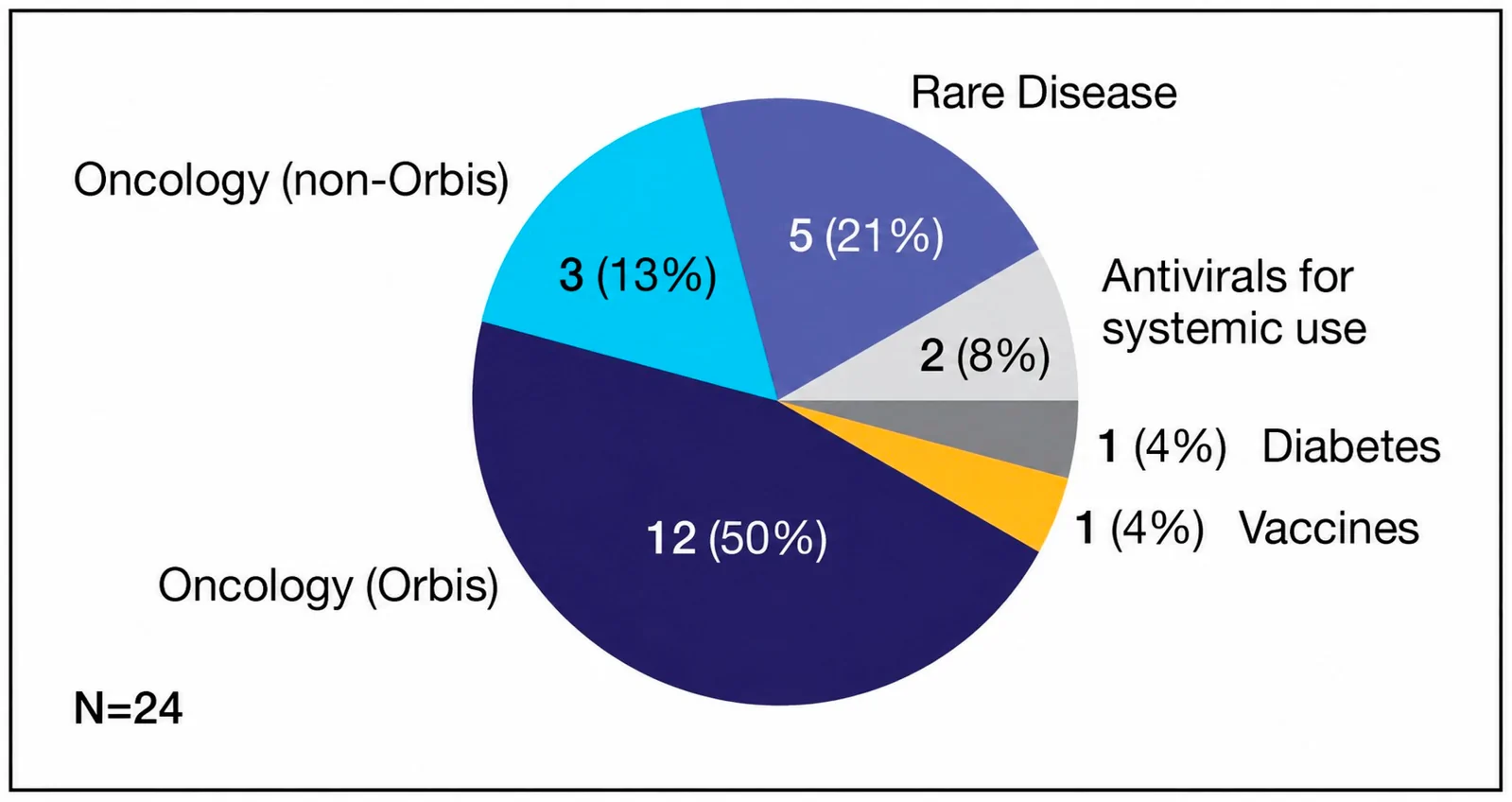
Health Canada Priority Review files approved in 2022, by category.

In 2022, 24 files received PR approval (Figure 1), of which oncology was the prominent category with 15 files (63%). Of the oncology files, 12 were reviewed under Project Orbis, and 3 were non-Orbis. Other categories included therapies for rare diseases (five files), antivirals (two files), one diabetes treatment and one vaccine (Figure A2).

The assessment of the overlap between Health Canada review pathways and Project Orbis participation showed that among the 117 files completed by HC in 2022, 85 (73%) were reviewed by HC under the standard review pathway, 24 (21%) under PR, and 8 (7%) under NOC/c. Twenty-one of the files were a part of Project Orbis, and these files overlapped with all three HC review pathways (standard, PR, NOC/c). The greatest overlap occurs among PR approvals, of which half are Orbis (Figure 1) (Table S1). Twelve PR non-Orbis drugs were identified, which are currently not eligible for existing accelerated access pathways (Table A1).

**Figure 2:**
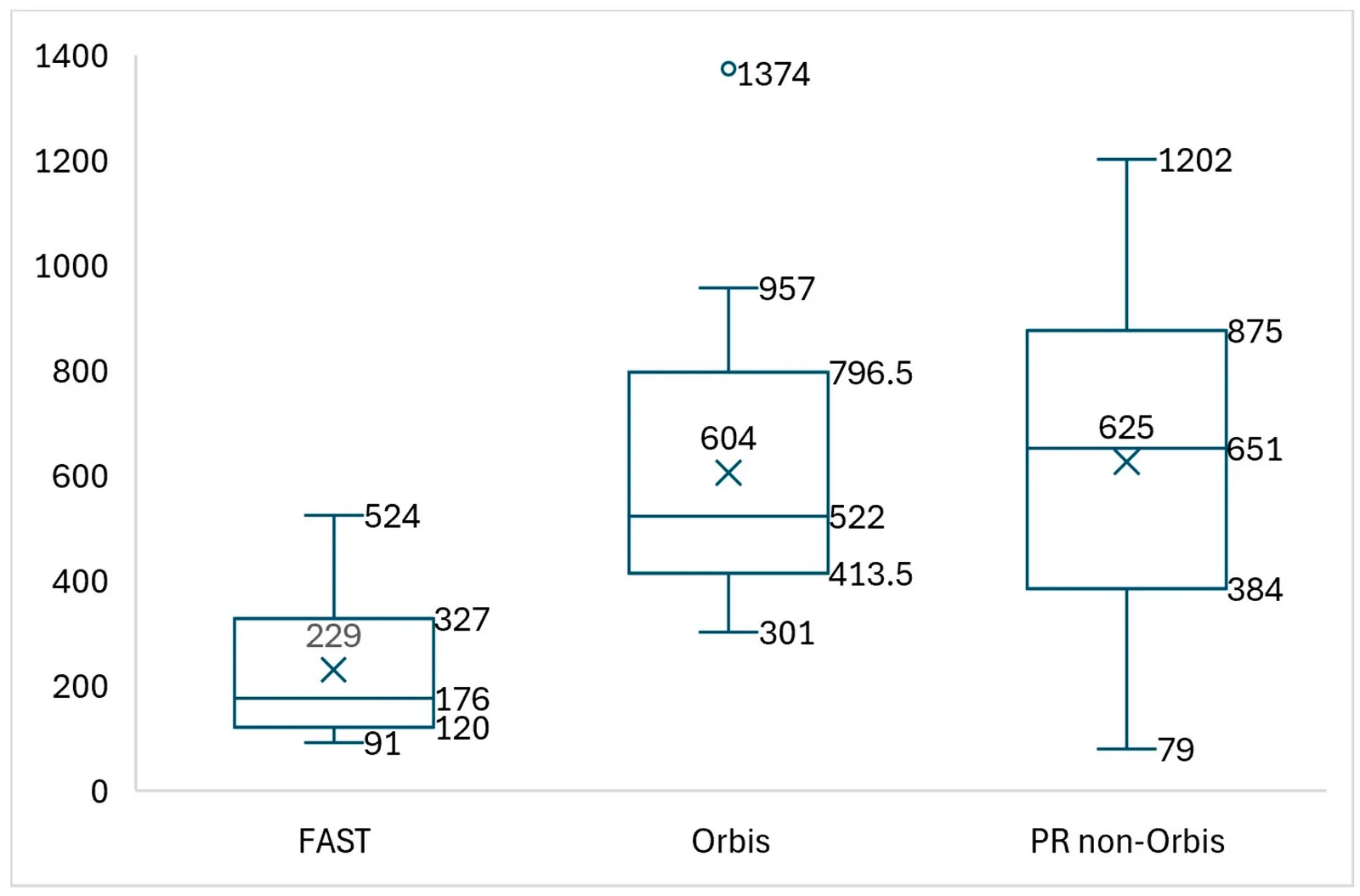
Time from NOC to Ontario listing (days). FAST = Funding Accelerated for Specific Treatments; PR = Priority Review.

**Figure A3:**
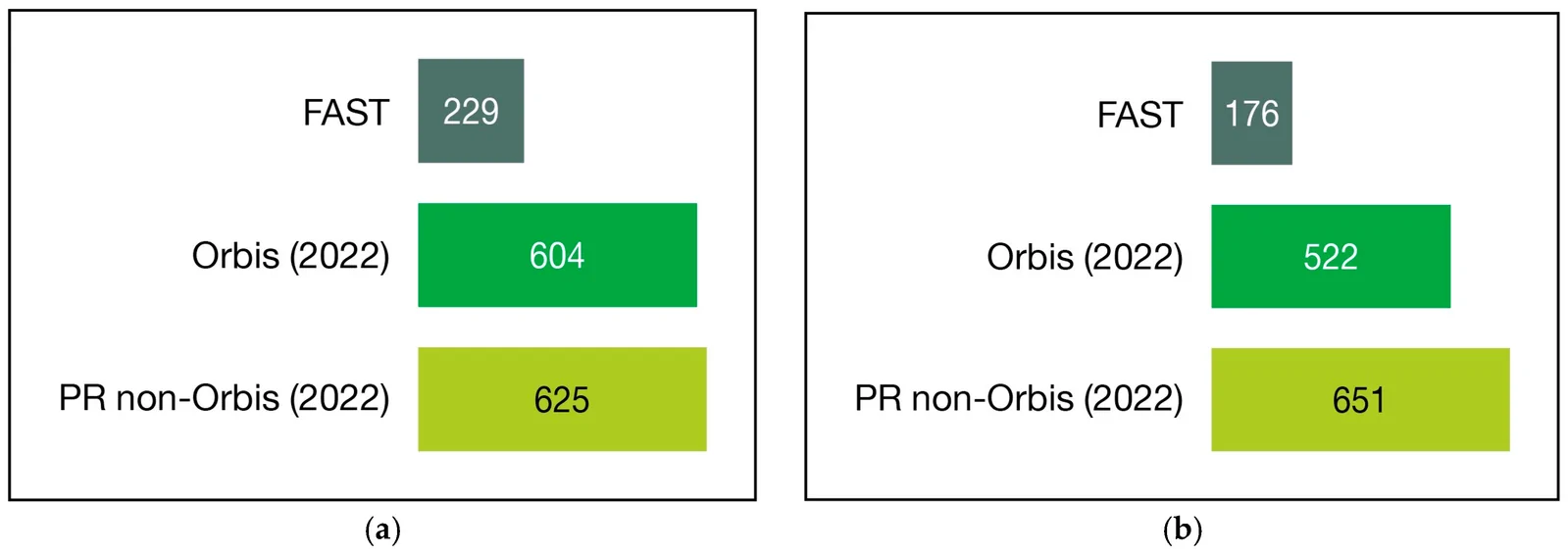
(**a**) Mean time from NOC to Ontario listing (days). (**b**) Median time from NOC to Ontario listing (days). FAST = Funding Accelerated for Specific Treatments; NOC = Notice of Compliance; PR = Priority Review.

**Figure A4:**
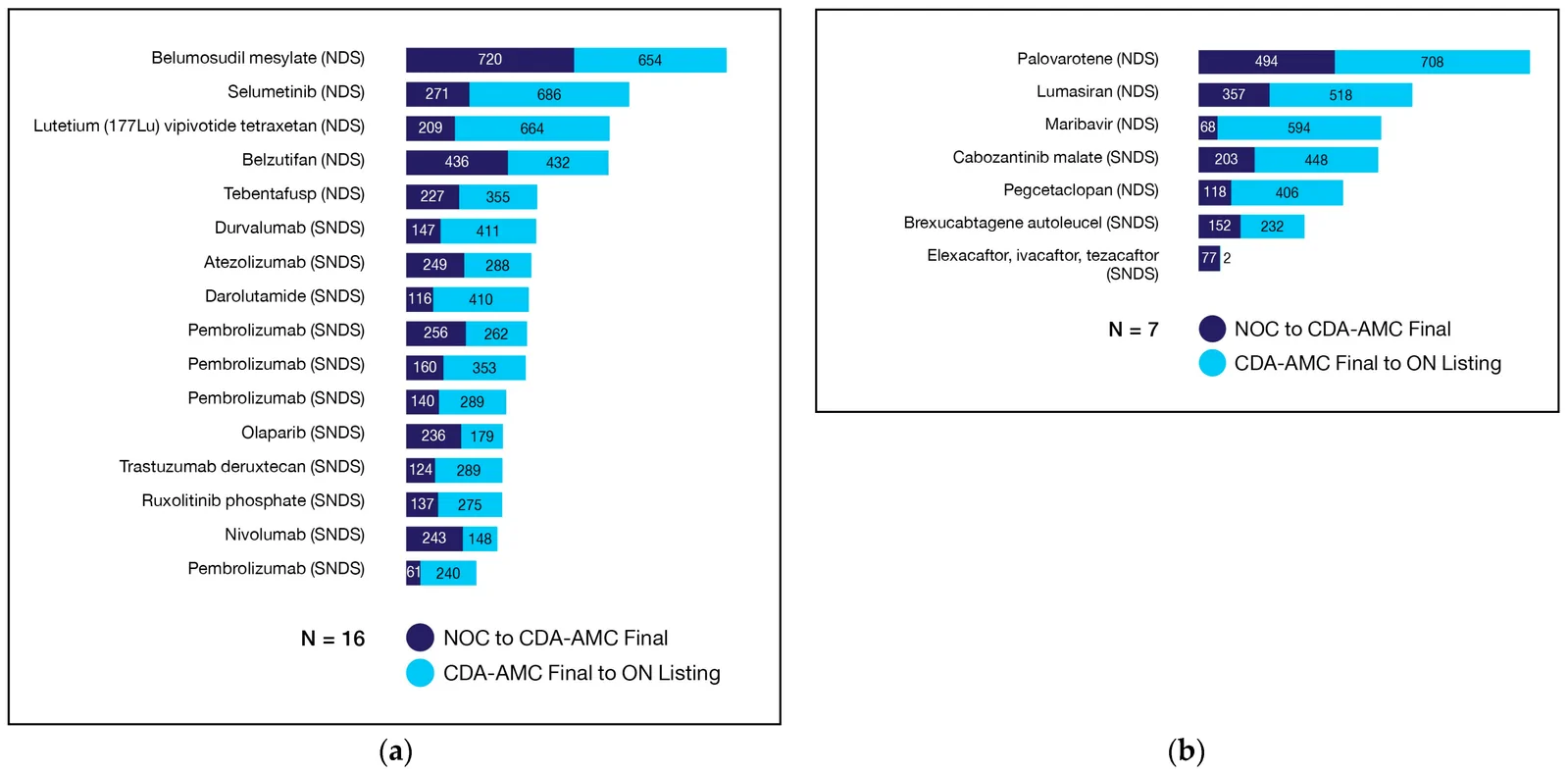
(**a**) 2022 Orbis files: Reimbursement timeline from NOC to Ontario listing with CDA-AMC final recommendation, for listed files (days). (**b**) 2022 PR non-Orbis files: Reimbursement timeline from NOC to Ontario listing with CDA-AMC final recommendation, for listed files (days). CDA-AMC = Canada’s Drug Agency; NDS = New Drug Submission; NOC = Notice of Compliance; ON = Ontario; PR = Priority Review; SNDS = Supplemental New Drug Submission.

Time-to-listing was assessed from NOC to Ontario formulary listing for Orbis and PR non-Orbis in the 2022 Health Canada data (Figure 2 and Figure A3a,b) (Table 1a and Table S1). At of the time of analysis, five Orbis drugs and four PR non-Orbis drugs had not received Ontario listing (Figure A4a,b). For Orbis, the mean time from NOC to Ontario listing was 604.18 ± 283.92 days (522 days median), and for PR non-Orbis, the mean time to listing was 625.29 ± 365.80 days (651 median). As shown in Figure 2, the time from NOC to Ontario listing for both groups is highly similar.

Nine drugs were found to be listed under FAST (Table S2). The mean time-to-listing was assessed from NOC to Ontario formulary listing for the FAST drugs, all of which are oncology therapies approved through Project Orbis, at 229.22 ± 140.50 days (176 days median) (Figure 2 and Figure A3a,b) (Table 1a and Table S1).

When comparing the mean NOC to Ontario listing timelines of FAST drugs with Orbis, FAST drugs were listed 62% faster. A similar acceleration is observed between the mean times of the FAST program and PR-non Orbis, with drugs listed 63% faster. (Figure 2 and Figure A3a,b) (Table 1a and Tables S1 and S2).

To determine whether the FAST program’s improvement over 2022 Orbis and PR non-Orbis approvals reflected more efficient CDA-AMC reviews, the interval from NOC to final CDA-AMC recommendation was compared between the two groups. FAST products had numerically shorter times from NOC to final HTA recommendation; however, the differences were not statistically significant (Table 1b), which suggests that most of the improvement occurred after CDA-AMC review and before listing. Whether pre-submission to CDA-AMC has an impact was not evaluated due to the lack of significance between FAST and PR non-Orbis in review times.

An analysis was carried out to calculate the volume of files in 2022 that would have been eligible for an accelerated access pathway based on current criteria, and the volume when including a potential PR pathway (Table A2). One file (1% of all approvals in 2022) was eligible for TLR-pTAP (actual volume), 21 files (18%) for FAST-ENP (estimated volume based on Project Orbis approved therapies), and 12 files (10%) for PR non-Orbis. This would result in a total of 29% of files being eligible for acceleration across all pathways.

## 4. Discussion

### 4.1. Regulatory Pathway Designation as a Primary Eligibility Criterion for Acceleration

Drug development has become increasingly sophisticated, with therapies targeting molecular alterations across multiple pathways. This complexity has increased the demands on regulatory review while also intensifying pressure to deliver timely patient access. In response, regulators have adopted collaborative review models such as Project Orbis, and expanded expedited review mechanisms, as seen with Health Canada’s PR and NOC/c. Since regulators evaluate either the full clinical package with standard and PR submissions, or all available early and promising data from NOC/c submissions, they are well positioned to identify products that warrant accelerated downstream access.

In this context, TLR-pTAP, FAST and ENP appropriately use regulatory pathway designation as a primary eligibility criterion. However, each approach has limitations: TLR-pTAP remains constrained by restrictive eligibility, while FAST and ENP may exclude innovative oncology therapies that did not enter Project Orbis. Drugs may not enter Project Orbis for several reasons unrelated to the potential high impact for patients of the treatment, including, for example, global filing and data timing considerations, resource constraints at the sponsor or regulator to leverage the pathway, or sponsor filing strategies [42].

To our knowledge, this is the first publication to measure the interval from NOC to provincial formulary listing, the point at which patients can formally access public reimbursement for a medication. As described in the results, the time from NOC to Ontario listing for drugs which received NOC in 2022 was similar across Project Orbis drugs and Priority Review non-Orbis drugs, consistent with prior Canadian reports. By contrast, time to listing for drugs in the FAST program was improved by approximately 60%, representing a substantial faster time to reimbursement. As such, findings from this research suggest that to avoid missing important opportunities for timely and equitable treatment access, accelerated reimbursement models could consider being expanded to include the Priority Review regulatory pathway.

### 4.2. Patient Equity Considerations

Current accelerated access pathways in Canada are largely oncology-focused, which has created inequity in access for other therapeutic areas. While oncology has been an appropriate starting point given its high disease burden and high unmet need, non-oncology drugs and the patients they treat are currently left behind. Extending a comparable accelerated funding mechanism to PR therapies, especially those supported by therapeutic advancement [9,43], could materially improve timely access for patients with serious illnesses beyond oncology.

Given the recent increase in rare disease therapies receiving Health Canada PR [8] (Table S3A,B), between 2021 and 2025 the proportion of oncology drugs decreased from 62% to 20%, whereas the proportion of orphan drugs for rare diseases increased from 53% to 80%, reaching 90% in 2023, together with rare disease therapies being the largest category in the PR non-Orbis group (Figure A2), an accelerated access pathway that includes drugs for rare diseases would be timely and align with Canada’s broader rare disease strategy. With the launch of the National Strategy for Drugs for Rare Diseases [44], a common list of drugs was selected for bilateral funding agreements [45]. Among these, two therapies, Oxlumo (lumasiran) and Sohonos (palovarotene), appeared in our analysis of 2022 PR non-Orbis drugs. For these products, the time from NOC to Ontario listing was 875 and 1202 days, respectively, indicating substantial delays in access despite recognized clinical importance (Table S1). If a FAST-like mechanism had been available for these therapies, patients with rare diseases may have gained access substantially earlier. A recent publication “Orphan Drug Approval in Canada, 1999–2022: A Cross-sectional Study” should be also taken into consideration [46].

### 4.3. Feasibility and Impact of Expanding Accelerated Access Pathways

From a feasibility standpoint, it is necessary to consider the appropriate number of files to accelerate, recognizing that not all therapies can or should be prioritized. If all PR drugs were to be included, this would result in approximately 29% of all drugs being eligible for an accelerated pathway. This may represent a reasonable proportion for acceleration, although pilot implementation will be needed to assess this in practice. It will also be important to measure the impact and associated effort of the accelerated pathways and to continue to refine processes. Another area of consideration for feasibility research is what the impact of expanding the acceleration eligibility would be for non-accelerated drugs.

### 4.4. A “Made-in-Canada” Approach for Canadian Patients

Although PR provides regulatory acceleration, half of PR therapies did not overlap with Project Orbis, which is the current eligibility criterion for FAST and ENP pathways. This highlights an opportunity for a more Canada-specific approach to accelerated pathways by including all PR therapies, either as a new pathway or within existing mechanisms, which may result in more equitable drug access. Given the Federal Government’s recent initiatives to remove interprovincial barriers and promote national strategies [47], the findings from this research could help clarify the opportunity for a unified approach to accelerating patient access across Canada.

### 4.5. Limitations

A major limitation of this research is that only one year, 2022, was selected for detailed analysis and comparison to the FAST program. The reason was to allow sufficient time to measure time to provincial listings, which historically could take up to three years. The mean time from NOC to Ontario listings for approvals in 2022 was 600 days or more, consistent with historical records [1], suggesting that data from 2022 could be generalizable and representative of time to reimbursement.

Another limitation of this research is that the number of patients to have benefitted from the FAST program was not assessed. To our knowledge this figure is not publicly available; however it would be helpful to understand the impact of this program.

### 4.6. Further Research

An important consideration is how “priority” is defined across stakeholders, including patients, payers, and manufacturers, and whether the regulatory definition of PR used by Health Canada aligns with their perspectives. Further research, specifically multi-stakeholder engagement, will be required to assess alignment on prioritization and implementation opportunities for an accelerated pathway for PR therapies.

Further research will also be required to assess the impact of the FAST and ENP programs to gain learnings from these pathways, as these are 3- and 2-year pilots, respectively. As shown in the time-to-listing results, the benefits of the FAST program have been achieved in the short term. In the long term, it remains to be seen whether FAST will continue to enable ongoing patient access, especially for higher-priced therapies. Given that the majority of CDA-AMC recommendations are to reimburse with clinical criteria and/or a price reduction in the range of 50–90% [48], files may experience challenges in the pCPA negotiation process, especially those with significant price reduction recommendations.

Another area for future research is to evaluate drug costs, financial implications, and potential risks associated with accelerated reimbursement pathways, including how payment mechanisms and subsequent price negotiations may affect provincial health system resources.

## 5. Conclusions

The Ontario FAST program is associated with substantially shorter times to public listing for novel oncology medicines. Extending a similar accelerated access pathway to therapies approved through Health Canada Priority Review, both in Ontario and in other provincial health jurisdictions, will improve access to novel therapies that address a high unmet need, and help establish a more timely and equitable drug access framework for Canadian patients.

## Figures and Tables

**Table 1:** (**a**) Statistical analysis of time from NOC to Ontario listing (days). (**b**) Statistical analysis of time from NOC to CDA-AMC final recommendation (days).

**(a)**					
**Status**	**Mean ± SD (95%CI)**	**Median (IQR)**	***p* Value**		
Orbis *n* = 16 ^#^	604.18 ± 278.82 (468, 741)	522 (416–654)	Orbis to PR non-Orbis	FAST to PR non-Orbis	FAST to Orbis
PR non-Orbis *n* = 7 *	625.29 ± 356.80 (341, 913)	651 (384–875)	0.547	0.008	0.001
FAST *n* = 9	229.22 ± 140.50 (137, 321)	176 (135–305)			
**(b)**					
**Status**	**Mean ± SD (95%CI)**	**Median (IQR)**	***p* Value**		
Orbis *n* = 16 ^##^	208.90 ± 186.98 (117, 300)	141 (123.5–274.5)	Orbis to PR non-Orbis	FAST to PR non-Orbis	FAST to Orbis
PR non-Orbis *n* = 7 **	209.85 ± 159.16 (95, 325)	184 (96–280)	0.98	0.28	0.12
FAST *n* = 9	151.2 ± 67.88 (113, 189)	113 (98–176)			

^#^ Five Orbis have not received Ontario listing; * Four PR non-Orbis have not received Ontario listing. ^##^ Five Orbis did not submit to CDA-AMC; ** Four PR non-Orbis did not submit to CDA-AMC. CI = confidence interval; FAST = Funding Accelerated for Specific Treatments; IQR = interquartile range; NOC = Notice of Compliance; pCPA = pan-Canadian Pharmaceutical Alliance; PR = Priority Review; SD = standard deviation. CDA-AMC = Canada’s Drug Agency.

## Data Availability

The original contributions presented in the study are included in the article; further inquiries can be directed to the corresponding author.

## Supplementary Materials

The following supporting information can be downloaded at: https://www.mdpi.com/article/10.3390/curroncol33090517/s1, Table S1. Health Canada 2022 approvals by analysis groups; Table S2. Drugs listed under the Ontario FAST pilot program. Table S3A,B. Health Canada Priority Reviews by therapeutic categories.

## Abbreviations

The following abbreviations are used in this manuscript:

CDA-AMC	Canada’s Drug Agency
CI	Confidence Interval
EAP	Exceptional Access Program
ENP	Early Negotiation Process
FAST	Funding Accelerated for Specific Treatments
FDA	Food and Drug Administration
HC	Health Canada
HPDD	Ontario Ministry of Health Drug Programs Delivery
HTA	Health Technology Assessment
INESSS	Institut national d’excellence en santé et en services sociaux
IQR	Interquartile Range
NDS	New Drug Submission
NOC	Notice of Compliance
NOC/c	Notice of Compliance with conditions
OECD	Organisation for Economic Co-operation and Development
pCPA	pan-Canadian Pharmaceutical Alliance
PR	Priority Review
pTAP	pCPA’s Temporary Access Process
SD	Standard Deviation
SNDS	Supplemental New Drug Submission
SUR	Submission Under Review
TLR	Time-Limited Recommendation

## Appendix A

**Table A1:** Priority Review non-Orbis files approved in 2022 not eligible for existing accelerated access pathways.

#	Brand Name	Medicinal Ingredient(s)	Category	Indication
1	Brukinsa	Zanubrutinib	Oncology	Marginal zone lymphoma (MZL)
2	Cabometyx	Cabozantinib malate	Oncology	Differentiated thyroid carcinoma (DTC)
3	Tecartus	Brexucabtagene autoleucel	Oncology	Acute lymphoblastic leukemia (ALL)
4	Sohonos	Palovarotene	Rare Disease	Fibrodysplasia (myositis) ossificans progressiva
5	Oxlumo	Lumasiran	Rare Disease	Primary hyperoxaluria type 1 (PH1)
6	Trikafta	Elexacaftor, ivacaftor, tezacaftor	Rare Disease	Cystic fibrosis (CF)—expanded indication
7	Kalydeco	Ivacaftor	Rare Disease	Cystic fibrosis (CF)—expanded indication
8	Empaveli	Pegcetaclopan	Rare Disease	Paroxysmal nocturnal hemoglobinuria (PNH)
9	Livtencity	Maribavir	Antivirals for systemic use	Post-transplant cytomegalovirus (CMV) infection/disease
10	Sunlenca	Lenacapavir sodium	Antivirals for systemic use	HIV-1 infection
11	Jardiance	Empagliflozin	Diabetes	Chronic heart failure (HF)
12	Vaxneuvance	Corynebacterium diphtheriae CRM-197 protein	Vaccines	Pediatric pneumococcal conjugate vaccine (PCV)

**Table A2:** Estimated accelerated access pathway eligibility for Health Canada files approved in 2022.

Accelerated Access Pathway	# of Files	% of Total Files Approved in 2022
1. TLR-pTAP (some NOC/c)	1	1%
2. FAST-ENP (Project Orbis)	21	18%
3. Priority Review Non-Orbis	12	10%
**Total**		**29%**

FAST-ENP = Funding Accelerated for Specific Treatments and Early Negotiation Process; NOC/c = Notice of Compliance with conditions; TLR-pTAP = pCPA’s Time-Limited Recommendation and Temporary Access Process.
