# Supplementary material for: Assessing the Opportunity for an Accelerated Access Pathway for Health Canada Priority Review Drugs: A Comparative Analysis with Ontario’s FAST Pilot Program
Source: Curr Oncol. 2026 Aug 29;33(9):517. doi: 10.3390/curroncol33090517 (PMC13605068; doi:10.3390/curroncol33090517)
Supplement: Supplementary file 1 [file curroncol-33-00517-s001.zip › Table S3.AB Health Canada Priority Reviews by Therapeutic Categories.pdf]

Supplemental Table 3A : Health Canada Priority Approvals by Categories from 2021 to 2025				
	**Name (generic)**	**Orphan Drug Designation***	**Therapeutic area**	**Antineoplastic + orphan drug designation**
**2025**	Olezarsen sodium	Yes	Lipid modifying agents	
	Sepiapterin	Yes	Other alimentary tract and metabolism products	
	Vamorolone	Yes	Corticosteroids for systemic use	
	Oritavancin diphosphate		Antibacterials for systemic use	
	Mirvetuximab soravtansine	Yes	Antineoplastic agents	✓
	Bulevirtide acetate	Yes	Antivirals for systemic use	
	Artesunate	Yes	Antiprotozoals	
	Teplizumab Y	Yes	Drugs used in diabetes	
	Omaveloxolone Y	Yes	Other nervous system drugs	
	Inavolisib		Antineoplastic agents	
	Delafloxacin meglumine		Antibacterials for systemic use	
**2024**	Meropenem trihydrate, vaborbactam	Yes	Antibacterials for systemic use	
	Trofinetide	yes	Other nervous system drugs	
	Exagamglogene autotemcel	Yes	Other hematological agents	
	Sotatercept	Yes	Antihypertensives	
	Vorasidenib citrate		Antineoplastic agents	✓
	Corynebacterium diphtheriae CRM-197 protein, pneumococcal conjugate serotype 33F, pneumococcal polysaccharide serotypes		Vaccines	

	Cabotegravir, cabotegravir sodium		Antivirals for systemic use	
	Olipudase alfa	Yes	Other alimentary tract and metabolism products	
	Metreleptin	Yes	Other alimentary tract and metabolism products	
**2023**	Fidanacogene elaparvovec	Yes	Antihemorrhagics	
	Odevixibat	Yes	Bile and liver therapy	
	Etranacogene dezaparvovec	Yes	Other hematological agents	
	Evinacumab	Yes	Lipid modifying agents	
	Maralixibat chloride	Yes	Bile and liver therapy	
	Erwinia L-asparaginase	Yes	Antineoplastic agents	✓
	Setmelanotide acetate	Yes	Antiobesity preparations, excluding diet products	
	Spesolimab	Yes	Immunosuppressants	
	Concizumab		Antihemorrhagics	
	Amifampridine	Yes	Other nervous system drugs	
**2022**	Pegcetaclopan		Immunosuppressants	
	Lenacapavir sodium	Yes	Antivirals for systemic use	
	Maribavir	Yes	Antivirals for systemic use	
	Lutetium (177Lu) vipivotide tetraxetan		Therapeutic radiopharmaceuticals	
	Tebentafusp	Yes	Antineoplastic agents	✓
	Belumosudil mesylate	Yes	Immunosuppressants	
	Lumasiran	Yes	Other alimentary tract and metabolism products	
	Palovarotene	Yes	Other drugs for disorders of the musculo-skeletal system	
**2021**	Enfortumab vedotin		Antineoplastic agents	
	Fostemsavir tromethamine		Antivirals for systemic use	

	Sacituzumab govitecan	Yes	Antineoplastic agents	✓
	Colchicine	Yes	Antigout preparations	
	Treosulfan	Yes	Antineoplastic agents	✓
	Elexacaftor, ivacaftor, tezacaftor	Yes	Other respiratory system products	
	Brexucabtagene autoleucel		Antineoplastic agents	✓
	Idecabtagene vicleucel		Antineoplastic agents	✓
	Cytarabine, daunorubicin		Antineoplastic agents	
	Risdiplam	Yes	Other drugs for disorders of the musculo-skeletal system	
	Zanubrutinib	Yes	Antineoplastic agents	✓
	Triheptanoin	Yes	Other alimentary tract and metabolism products	
	Azacitidine		Antineoplastic agents	

* https://pmc.ncbi.nlm.nih.gov/articles/PMC10924572 & https://aamac.ca/wp-content/uploads/2017/11/2013_Orphan_Drug_Framework.pdf#1%231

Supplemental Table 3B: Summary of Health Canada Priority Reviews by Therapeutic Categories from 2021 to 2025

Year of Approval	2021	2022	2023	2024	2025
**Total approvals**	13	8	10	9	10
**Orphan Drug (OD) N (%)**	7 (54)	6 (75)	9 (90)	6 (67)	8 (80)
**Oncology N(%)**	8 (62)	1 (12.5)	0	1 (11)	2 (20)
**OD + Oncology N(%)**	5 (38)	0	0	0	1 (10)
